# Empathy in Psychoanalysis and Medical Education - what can we learn from each other?

**DOI:** 10.1186/s12909-017-0907-2

**Published:** 2017-05-02

**Authors:** Henriette Löffler-Stastka, Felicitas Datz, Karoline Parth, Ingrid Preusche, Xenia Bukowski, Charles Seidman

**Affiliations:** 1 Department for Psychoanalysis und Psychotherapy and University Program for Psychotherapy Research, Medical University of Vienna, Währingerstraße 18-20, 1090 Vienna, Austria; 2 Medical University of Vienna, Teaching Center, Postgraduate Program, Vienna, Austria; 3 University of Veterinary Medicine, Teaching Center, Vienna, Austria; 4 International Psychoanalytic University, Berlin, Germany; 5 Emory University, Atlanta, USA

**Keywords:** Empathy, Psychoanalysis, Medical education, Medical curricula, Training, Nonverbal communication

## Abstract

**Background:**

Several research areas, including medical education (ME), focus on empathy as an important topic in interpersonal relationships. This focus is central to the use of communication skills related to empathy and even more crucial to provide information in a way that makes patients feel more involved in the treatment process. Psychoanalysis (PA) provides its initial concept of empathy based on affective aspects including findings from neuroscience and brain research. Enhancing cooperation between ME and PA can help to integrate both aspects of empathy into a longitudinal training program.

**Discussion:**

The condition of psychoanalytic empathy definitions is the understanding of unconscious processes. It is important to primarily attend especially the dominant ﻿affects towards the patient before interpreting his or her behaviour, since in explaining the emerging affects, the analyst has to empathize with the patient to understand the (unconscious) reasons for its behaviour. A strong consideration of nonverbal communication, clinical perceptions, intuitive interaction, contagion-like processes and their implementation and empowerment in medical and therapeutic curricula is one way of beneficially using interdisciplinary approaches to yield empathy in clinical interaction.

**Conclusion:**

Established methods of PA, like training of containment, reflective functioning, affective holding and giving meaningful interpretations in accordance with countertransferential and transferential aspects may help to put a focus on the clinican-patient-interaction and the preservation of the physicians’ (mental) health. In consequence of the discussion of various training methods that take the theoretical and practical concepts of empathy into account, we aim for an implementation of the named methods in the medical curricula.

## Background

Several research areas, including medical education (ME), focus on empathy as an important topic in interpersonal relationships. This is important for two main reasons in the medical encounter: First, to use communication skills related to empathy in order to collect more complete information to correctly diagnose diseases is often intertwined with empathy. Secondly and more importantly, empathy is crucial in order to provide information in a way that makes patients feel more involved in the treatment process.

In current research empathy is no longer seen as an either affective or cognitive capacity, but as a complex interplay of emotional, cognitive, and behavioral processes [1]. Many ME approaches tend to highlight the cognitive aspects of empathy [2]. Joseph Betancourt [3] on the other hand stressed the importance of sociocultural factors in determining clinical decision making and outcomes. This also hints at the importance of the student’s social education, implying that his or her entire upbringing could have clinical consequences. Psychoanalysis (PA) provides its initial concept of empathy based on affective dynamics in a person’s early years of life, including findings from (social) neuroscience and brain research.

Focusing on sociocultural factors, ME is encouraged to take psychodynamic aspects into account, taking psychological, developmental psychological and psychoanalytical concepts on empathy into account. An integration of psychoanalytical concepts can help to capture all aspects of empathy and implement them in a longitudinal training program, by widening the focus and using assessment methods in the workplace.

### Empathy and psychoanalysis

In PA there are several approaches regarding the concept of empathy [4]. The common aspect of all definitions, however, is the understanding of unconscious processes. Widlöcher [5] has tried to specify the psychological mechanisms operating in this complex form of intuitive understanding, specifically emphasizing the role of identification and inference. In general, empathy originates in affective processes, but is gradually elaborated within cognitive development. Therefore, empathy is understood as the affective experience of another person’s emotional state, as well as its recognition and understanding [6]. Since affective development is most intense during infancy, the caregiver’s ability for empathy becomes particularly important for the human being’s further development [7–10].

A child’s development of empathy is influenced by various components, such as genetics, facial mimicry and imitation, subserving areas of the brain such as the mirror neuron system and the limbic system, child temperament, parenting factors such as warmth, parent-child synchrony [11].

In summary, the ability of empathy is closely related to the development of affect and the differentiation between self and other. Its further actions are accordingly reflexively and purposefully [12]. In PA, object relations theory refers to identification processes and describes that the specific individual quality, by that we perceive people and interact with them, is restructured by our earliest relationships. Object relations theorists have made important contributions to our understanding of empathy (see e.g. [13, 14]). Their works illustrate the complexities involved in processes such as the projection of affects into others, which can also be an anti-empathic factor. Each (part-) object relationship is associated with affects and physiological impulses. Emotions and self-development happen at the same time. In its developmental period, in which the complex neuronal association fibres and areas are differentiated and associated step by step to higher cortical areas, the infant experiences individual affective events, shortly reaching consciousness and representing a self-experience. Thus, the self- development and (part-) object formation is always associated with affects, as they are always the primary driving force and therefore involved in the formation of self-elements [15]. The development of self- and object- representations helps the infant to independence from the objects real presence. This enables an idea about the own emotional situation and of one other's.

### Psychoanalytical concepts and clinical implementation

For the clinical encounter, for diagnostic purposes, clinical reasoning processes and especially for clinical authentic care, empathy helps to understand the patients’ concern and maintaining the clinician’s ability to think, reconsider and decide. However, empathy is far more complex when it comes to the therapeutic setting, since affects are more exposed than in everyday life [16]. Empathy can help the clinician to observe affects fearlessly and regulate them, with regard of the therapeutic aim to initiate a change in patient’s relating to his affects. Within empathic clinical work, mentalized affectivity [17] is relevant to understand unconscious communication processes, it is more precisely to identify and mark the dominant affective signal/concern, and to modulate process and contain these signals. The contained and metabolized affect has to be expressed to the patient in a way that is often described as “metabolized”. This digestion process requires the clinician to be familiar with his own subjective experience, requiring a high level of self-reflexivity. Containing the patient’s externalized affects enables the clinician to understand projected signals.

Aspects of this process are demonstrated most clearly in projective identification. It’s about the externalization of an internal object relationship in which the clinician is forced into a specific role by the patient. Contrary to this, he or she uses empathy as a brief identification process to observe the internal and expressed affect of the patient, but with no enforced role [18]. If the first aspect of this process is transference, the second will be the counter-transference. The last one contains two reactions: complementary and concordant. Complementary counter-transferences are affects that emerge during the treatment and are associated with meaningful objects. Concordant counter-transferences are basically empathic responses of experiences with patient’s emotional situation [18]. It is important to primarily attend especially the negative counter-transference before interpreting the transference. Because in explaining the emerging affects, the clinician has to empathize with the patient in order to understand the (unconscious) reasons for its behaviour.

### Empathy and medical education

In Medical Education (ME), the impact of empathy on interpersonal relationships has been strongly focused upon. However, many concepts used in ME define empathy primarily cognitively, with little importance assigned to the affective components [19, 20]. As discussed, the quality of the communication between clinicians and patients is closely related to the grade of care and patient contentment [21]. During medical education, translation of common knowledge into practice is often difficult to reconcile for many students from what they learned to the (future) professional field. The issue lies in finding a balance between distance and empathy for many students. Too much distance easily creates a mechanistic processing of the patient and makes it more difficult to relate to his or her affective situation. This struggle is further complicated by the increasing pressure to see more patients within scant time, which leads to amplified stress and can result in reduced feelings of empathy [22].

The addressed challenges call for anchoring of sophisticated empathy concepts and training tools in ME. Teachers can support students to transport empathy authentically into practice by drawing attention to the affective components of the clinician patient interaction as valuable resources.

During medical training empathy should be incorporated by training clinician patient communication encounters. Although didactic approaches based on a theoretical concept may form a solid basis, effective communication skills are displayed less in knowledge of theoretical concepts than in acting itself. Initiating encounters with standardized patients in early years of medical training and at a later stage at the workplace, assessment and immediate feedback helps the (prospective) physician to experience him or herself in interaction with the patient, but also question it critically [23].

### Aspects of clinical empathy research

Clasen et al. [24] evaluated the feasibility of training empathic communication and its consequences on the students’ empathy behaviour in talking to patients. This was done in the light of the fact that high empathy in the clinician-patient-relationship leads to a higher patient satisfaction, better compliance and better clinical outcome. The study revealed that the potential empathy chances (PEC) do not become explicit empathy chances since patients often stick to a factual level of communication. It needs to be clarified whether the causes lie in the patient, external parameters or in the conversational skills [24].

In order to foster high quality ME it appears reasonable to study differences in the clinical gaze, awareness and “contagion-like” processes between highly practiced clinicians (e.g. with more than 15 years of experience), as clinical reasoning consist of both, intuitive and analytical components [25]. The distinction between more and less experienced clinicians will help to highlight differences in their clinical judgment and the promptness of their empathic relationship building with their patients.

This becomes even more relevant when looking at recent studies on the heterogeneity of empathy in medical students [26].

## Discussion

In current empathy research one of certain approaches potter at “contagion-like” process. “Contagion-like” describes “the tendency to automatically mimic and synchronize facial expressions, vocalizations, postures, and movements with those of another person and, consequently, to converge emotionally” [27]. Higher-level cognitive processes in empathy on the other hand describe the act of willingly taking the perspective of another person [28].

Emotional contagion describes an unintentional imitation of an observed person’s facial expression, tone of vocalization, posture, and movements, which consequently leads to an emotional state in the observer similar to the one of the target [27]. Emotional contagion can be used as a tool in order to enable the understanding of the other’s emotion [9] in the everyday life but also in patient clinician interaction. In the latter case emotional contagion should not be acted out from the clinician’s side, but perceived and used in order to understand transference and counter-transference processes. In spite of enabling clinicians to train their perception and awareness for empathic processes an enhanced focus on the perception of nonverbal elements and processes within the interaction is crucial. One way to meet this objective is to specifically tutor nonverbal perception by means of video material. A conference with the therapists, subsequent to the recorded sessions, about the video material is ideal to analyze nonverbal perception [29]. As stated, nonverbal perception is often unconscious, pertinent and intuitive, running the risk of not being caught in research and awareness, although necessary when it comes to understanding and improving theoretic and clinical concepts of empathy.

Due to their good measurability several studies engage in the analysis of facial expressions and their importance in the therapeutic process. (see e.g. [30–32]) found a link between the synchronized smiling of therapist and patient and the treatment success. Merten and Benecke [33] showed that mimic synchronicity of therapist and patient is highly correlated to the treatment outcome. Unconscious interaction patterns are implemented through nonverbal signals. If the therapist manages to refuse these relationship cues, the implementation of pathological relationship patterns can be prevented. The higher the affective neutrality the greater the treatment success [33]. These and similar findings [34] once again point out the meaningfulness of training clinicians’ perception of nonverbal processes in order to establish and improve empathic relationships between clinician and patient.

Following studies on mental processes, an increasing body of evidence is yielded showing that disease patterns are stored in “frames,” “clinical scenarios,” “semantic networks/qualifiers,” or “illness scripts.” Repeated presentation and exercising of clinical cases is known to be crucial for an efficient learning process [35]. A strong consideration of nonverbal communication, clinical perceptions, intuitive interaction, contagion-like processes and their implementation and empowerment in medical and therapeutic curricula is another way of beneficially using interdisciplinary approaches to bring forward empathy in clinical interaction.

Constantin Stanislavski’s technique [36], known simply as “The Method” was revolutionary for theatre acting. Simply put, the method encouraged a psycho-physical union in which actors were required to mimic not only the affective states of their character, but their physical movements as well. Although initially developed for the stage, the method has since come to have a broad range of implications and uses across a number of disciplines, including the training of medical students. The question “What would motivate me, the actor, to behave in the way the character does?” forms the basis by asking the actor to replace the play’s circumstances with his or her own, the substitution ([37], 221).

Medical school all over the world implement training in interaction with patients, especially the training of techniques to convince a patient that a given course of treatment is the best option. These techniques almost invariably involve understanding and accommodating the patient’s emotions, especially their fears and anxieties. Medical students are usually trained with actors pretending to be patients, and the simulation typically ends when a ‘patient’ can be convinced to adopt the best course of treatment. While serious efforts have been given to training patient interaction, it stands to reason that the quality of the actor playing the patient is of the utmost pedagogical importance. How can the future doctor adapt to the fluid dynamics of patient interaction if the actor playing the patient is simply reading from a script? Genuine countertransference of the doctor is contingent on a believable display of patient transference.

Consequently, in order to effectively train future doctors in the vital and difficult art of patient interaction, actors who are better able to embrace the roles of the patients they are playing are necessary.

To validate the therapist’s efforts, PA uses Balint groups and supervisions, to train reflective components, mentalizing and containment in order to understand the patient’s concerns and thereby optimize the treatment. This specific form of supervision might be just as suitable for medical training as it supports the clinician to reflect on the interaction dynamic. Another possibility to validate these competences and their development are through reflective writing or (student) diaries [38].

One of the most often cited instruments in ME is the Jefferson Scale of Physician Empathy, which focuses on aspects of affect, for instance “Physicians’ understanding of their patients’ feelings and the feelings of their patients' families is a positive treatment factor.” The use of this instrument or measuring the general intention to show empathy according to the theory of planed behaviour in ME is especially helpful when it comes to investigating the stability or changes of empathy among medical students and residents throughout their training.

## Conclusion

The doctor-patient relationship is almost indistinguishable from what it was 50 years ago. Modern medicine places a greater emphasis on working with the patient rather than paternalistically prescribing a course of treatment which the patient is expected to follow.

Taking actual complex concepts of empathy into account training qualitative interpersonal relationships can yield to a high quality in the patient-clinician-relationship. Cognitive empathy training, often focuses on communication skills trainings, mostly with standardized patients, highlighting methods to convey empathic understanding with certain techniques (e.g. active listening). These cognitive components of empathy that are translated into training of communication skills techniques are necessary as they form the basis of effective communication.

Empathy through the psychoanalytic lens is the ability to recognize and empathize with other people’s feelings. Both the clinician and the physician have to use their communication skills to show empathy towards the patient. Medical students’ skills are trained during their medical education. However, the handling and using of the affective components of empathy are often not discussed and trained sufficiently. PA has established methods to use (affective) empathy with both patients and clinicians. One of them is mentalization, a mechanism that can be described as the sense-making of each other when it comes to subjective and mental processes. It is easy to assume that mentalization can be a problem in most mental issues [39]. Thus, it is even more important to not only work on the patient’s ability to mentalize in treatment, but beforehand focus and train the clinician’s capability of mentalization, and its use in working with patients. On the basis of manifold research, it is evident that mentalizing must be embodied during the pre-language period. Thereby, the quality of the nonverbal interaction between the parent and the infant is underlying the parent’s quality of mentalizing of the infant’s experience [39]. This is in line with the concept of Parental embodied mentalizing (PEM)—defined as the “parental capacity to (a) implicitly conceive, comprehend, and extrapolate the infant’s mental states (such as wishes, desires, or preferences) from the infant’s whole-body kinesthetic expressions and (b) adjust one’s own kinesthetic patterns accordingly ([40], 187).

We suggest to widen the scope of Case Based Learning (CBL) through simulated patient training with “patients” giving feedback from the concise patients view; ample data is given showing CBL to be an enjoyable and motivational didactic tool and effective in assisting in the expansion of declarative to procedural knowledge in academia [41]. Although a plethora of studies employ multiple choice questions (MCQs) in their investigation, few studies measure CBL, attenuated changes in student’s procedural knowledge in practice [41, 42] or employ comparison or control groups in isolating causal relationships [43–45]. Schwartz et al. [46] compared the efficacy of SP training which employed electronic mannequins to CBL in medical students. Although no significant difference in performance was described, surrogate parameters were measured in OSCE scores, thereby evaluating procedural knowledge in practice.

Further, [47] demonstrated the effectiveness of psychoanalytic case supervision for teachers augmenting their mentalization skills.

Reflective portfolios for instance, prove to be of value when it comes to assessing students’ problems during their training [48]. Furthermore, established methods of PA, like containment, reflective functioning, affective holding and giving meaningful interpretations in accordance with (counter−) transferential aspects can help to lay a focus on the preservation of the physicians’ (mental) health. As to our knowledge, mental hygiene is not formally taught or regularly facilitated in medical curricula.

As stated, videotaped sessions are advantageous not only in training students’ affect perception but also in enabling a more holistic understanding of the verbal and nonverbal, conscious and unconscious processes in the clinician-patient interaction. A conference with the students, subsequent to the video-taped interactions, dealing in terms of verbal and nonverbal behaviour, is ideal to analyze dynamics and train their empathic understanding.

In consequence of our discussion of various training methods that take the theoretical and practical concepts of mentalization and empathy into account, we hereby aim for an implementation of the named methods in the medical curricula.
